# Direct and Indirect Serum Markers of Liver Fibrosis Compared with Transient Elastography among Women in the Women’s Interagency HIV Study

**DOI:** 10.4172/2155-6113.1000446

**Published:** 2015-04-10

**Authors:** Seble Kassaye, Ying Li, Gregory Huhn, Marion G Peters, Audrey L French, Phyllis C Tien, Bruce Luxon, Michael W Plankey

**Affiliations:** 1 Department of Medicine, Georgetown University Medical Center, Washington, District of Columbia, USA; 2 CORE Center/Department of Medicine, Stroger Hospital of Cook County, Chicago, Illinois, USA; 3 Department of Medicine, University of California, San Francisco, California, USA; 4 Medical Service, Department of Veterans Affairs Medical Center, San Francisco, California, USA

**Keywords:** HIV, HCV, Biomarkers, SHASTA, APRI, Transient elastography, WIHS, Women

## Abstract

**Objective:**

The aim of this study was to determine the test characteristics of direct and indirect biomarkers for liver fibrosis compared with transient elastography (TE) among a group of human immunodeficiency virus (HIV)-infected and uninfected women with or without Hepatitis C virus (HCV) infection.

**Methods:**

Women enrolled in the Women’s Interagency HIV Study (WIHS) from Washington DC, San Francisco, and Chicago with a body mass index (BMI)<35 underwent liver stiffness measurement using TE between October, 2010 and September, 2012. Serum samples were tested for hyaluronic acid to calculate the SHASTA and aspartate aminotransferase to platelet ratio index (APRI). Receiver operator characteristics (ROC) of significant liver fibrosis (liver stiffness ≥ 7.1 kPa by TE, correlating with a METAVIR fibrosis score of F2-F4) predicted by SHASTA and APRI were compared.

**Results:**

Among 308 women, the median age was 48 years, BMI was 25.6, 67% were non-Hispanic black, 27% HCV+, and 78% HIV+. The overall prevalence of significant liver fibrosis was 20%, and among HIV+ women, 22%. Overall, there was no statistically significant difference in the area under ROC curve (AUROC) between SHASTA and APRI relative to significant fibrosis by TE. Among HCV+ women (with or without HIV), the AUROC ranged from 0.70–0.73 for both the SHASTA and APRI compared to TE. Both SHASTA and APRI were associated with significant misclassification with a false negative rate of 33–40% for significant fibrosis compared with TE among women with HCV infection, with or without HIV.

**Conclusion:**

Both the SHASTA and APRI, direct and indirect serum biomarkers of liver fibrosis respectively, are comparable at detection of significant liver fibrosis among women with HCV infection, regardless of HIV status. However, there was a high false negative rate in detection of significant liver fibrosis of up to 40% which is a significant limitation of use of these biomarkers.

## Introduction

Recent advances in hepatitis C (HCV) treatment have led to the availability of anti-viral agents with high rates of sustained virologic response (SVR) with cure rates for HCV infection exceeding 90–95% [1,2]. While the ease, tolerability, and efficacy of newer all-oral HCV treatment regimens may eventually lead to early treatment, the expense of these newer medications, along with limited health care resources, often result in treatment decisions being based on degree of liver fibrosis. Determination of the degree of liver fibrosis among individuals with chronic HCV thus remains an important component of care to determine urgency for treatment of HCV, and to identify the presence of advanced liver disease and cirrhosis that mandates screening for hepatoma and presence of gastroesophageal varices [3–5]. However, there is decreasing enthusiasm for direct histologic visualization of the liver parenchyma via liver biopsy given the potential, albeit rare, serious complications including significant bleeding.

Non-invasive biomarkers are being used to evaluate liver fibrosis. Some biomarkers are altered secondary to the liver parenchymal distortion associated with fibrosis and serve as indirect markers of liver dysfunction while others provide a direct measurement of the degree of extracellular material deposition. Combinations of indirect biomarkers such as the aspartate aminotransferase (AST) and platelet count can be used to calculate the AST to platelet ratio index (APRI). Use of the APRI as a biomarker for liver fibrosis is appealing as the component lab tests are performed routinely in the management of HIV and HCV and it has a high negative predictive value of 0.87 for significant fibrosis (METAVIR stage F ≥ 2) [6]. Potential limitations of the APRI for identifying liver fibrosis includes variability in test characteristics with worsening immune status [7], due to either the direct effects of HIV or commonly used antiretroviral medications on these measurements independent of liver fibrosis. Hyaluronic acid (HA) has been used as a direct marker of liver fibrosis, as its production is associated with the deposition of the matrix that contributes to fibrosis. Among HIV/HCV co-infected individuals, HA levels have been associated with a high negative predictive value in predicting the absence of cirrhosis, and a high positive predictive value for identifying minimal fibrosis when combined with albumin and AST as part of the SHASTA index [8].

Vibration controlled transient elastography (TE) uses sound waves to measure liver stiffness, and has been in use in Europe and Asia for a number of years, and was approved by the U.S. Food and Drug Administration in 2013. This non-invasive test uses ultrasound technology to determine liver stiffness, and has been shown to have a high negative predictive value for detection of cirrhosis [9], including in patients with HIV [10,11]. Studies of these non-invasive methods among HIV/HCV co-infected individuals have small sample sizes, and include mostly men [10,12]. In this study, we compared the test characteristics of the SHASTA index and APRI for determination of significant fibrosis measured by the TE device, TE: Fibroscan^®^ among women with HIV/HCV coinfection, HIV monoinfection, HCV monoinfection, and those with neither infection.

## Materials and Methods

### Participants and procedures

The Women’s Interagency HIV Study (WIHS) is a multicenter prospective cohort study established in 1994 to investigate the progression of HIV infection. A total of 3,766 women (2,791 HIV-seropositive and 975 HIV-seronegative) were enrolled in either 1994–1995 (n=2,623) or 2001–2002 (n=1,143) from six United States cities (New York (Bronx and Brooklyn), Chicago, Los Angeles, San Francisco and Washington DC) [13]. Every six months, participants complete a comprehensive physical examination, provide blood specimens for CD4+ cell count and HIV RNA determination, and complete an interviewer-administered questionnaire, which provides data on demographics, medical history, and specific combination antiretroviral therapy (ART) use. The WIHS uses a standard definition of ART adapted from the Department of Health and Human Services/Kaiser Panel guidelines [14].

TE has been used to measure liver stiffness at the San Francisco site since October 2010 and at the Chicago and Washington DC sites since April 2011. Exclusion criteria were as follows: 1) current pregnancy; 2) current BMI (body mass index)>35 kg/m^2^; 3) detectable serum hepatitis B surface antigen; 4) evidence of decompensated cirrhosis (ascites, hepatic encephalopathy, esophageal varices); 5) evidence of severe renal insufficiency (defined as estimated glomerular filtration rate<30 ml/min); 6) acute hepatitis, hepatitis flare or cholestasis (due to interference with liver stiffness measurement); 7) known chronic liver disease (primary biliary cirrhosis, hemochromatosis, autoimmune hepatitis, or hepatocellular carcinoma) [15]. Using these criteria, TE was performed on 377 WIHS participants with a study visit between October, 2010 and September, 2012. Among them, 170 were at San Francisco site, 94 at Chicago site, and 113 at Washington DC site.

During the WIHS participant’s semiannual visit, data regarding their age at the visit, race/ethnicity (white non-Hispanic, black non- Hispanic, or other), BMI, history of injection drug use, alcohol use, and smoking were collected. HIV status was defined as “positive” when participant had a positive HIV enzyme immunoassay (EIA) result confirmed by Western blot. HCV status was “positive” when participant had a positive HCV EIA result confirmed by detectable HCV RNA. For analysis purposes, history of any illicit injection drug use was coded as a “yes” or “no” categorical variable; alcohol use was coded as “abstainer” (i.e., 0 drinks/week), “light” (<3 drinks/week), “moderate” (3−13 drinks/week), or “heavier” (≥ 14 drink/week), and smoking status as “never”, “current”, or “former”.

### Liver stiffness measurement

TE has been developed to quantify the degree of liver fibrosis by measuring liver elasticity non-invasively. Liver elasticity is measured by producing a relatively low speed elastic wave (~1 m/sec) and then assessing its propagation velocity with high speed ultrasound. The speed of wave propagation through the liver is directly correlated with the square root of the elastic modulus or tissue stiffness and reported in kilopascals, kPa) [1–3,16–19]. In this study, experienced clinicians were trained and certified to perform TE. A TE measurement was considered “reliable” when it satisfied the following criteria: (i) at least 10 valid test results were obtained at a single visit; (ii) >60% success rate (i.e., number of valid tests divided by the number of total tests); (iii) the interquartile range (IQR) of the last 10 valid test results was <30% of the median of last 10 test results. Liver stiffness was determined by the median of the last 10 valid test results for a reliable TE measurement according to the manufacturer’s instructions.

Liver fibrosis stage was derived using validated cutoffs [4]; a liver stiffness value <7.1 kPa corresponded to the METAVIR histologic scoring system, F0–1; ≥ 7.1 kPa and <9.5 kPa to F2; ≥ 9.5 kPa and <12.5 kPa F3; ≥ 12.5 kPa F4. F0−1 was considered as no/little fibrosis and F ≥ 2 as significant fibrosis.

### Serum markers

Participants had standard laboratory assessments performed by licensed clinical laboratories from serum samples collected during their visits. These assessments included CD4+ cell count, HIV RNA level, HCV RNA level, hyaluronic acid (HA), alanine aminotransferase (ALT), aspartate aminotransferase (AST), albumin, and platelet count.

SHASTA index and APRI were calculated with the following formula [5,8,12]:

$$SHASTA=\frac{\text{exp}(\text{risk score})}{1+\text{exp}(\text{risk score})},$$

risk score=−3.84+1.7 × (1 if HA in 41–85 ng/mL, 0 otherwise)+3.28 × (1 if HA>85 ng/mL, 0 otherwise)+1.58 × (1 if albumin<3.5 g/dL, 0 otherwise)+1.78 × (1 if AST>60 IU/L, 0 otherwise)

$$APRI=\frac{AST}{\text{upper normal limit of}AST}\times \frac{100}{\text{platelet count}}$$

### Statistical analysis

Demographics and clinical characteristics were described by HCV and HIV status. Since the numerical variables did not follow normal distribution, Wilcoxon-Mann-Whitney test (a non-parametric analog to the independent samples t-test) was performed to compare the numerical variables by HCV status and a Chi-square or Fisher exact test was used for the categorical variables. The diagnostic performance of SHASTA and APRI was assessed by using receiver operating characteristic (ROC) curves and compared by HCV status overall and among HIV+ women, respectively. ROC curves were obtained using simple logistic regression models with liver stiffness (i.e., F ≥ 2 vs. F0−1) as the outcome and SHASTA or APRI as the continuous predictor. The most commonly used index of accuracy is the area under the ROC curve (AUROC), with values close to 1.0 indicating high diagnostic accuracy [4]. A Chi square test was run to evaluate the difference of the AUROCs between SHASTA and APRI. Optimal cut-off values were chosen to maximize the sum of sensitivity and specificity [4], and false positive/negative rates were computed for these cut-off values.

## Results

The study sample was composed of 308 after the exclusion of 44 women without a “reliable” TE measurement and 25 with missing lab data to calculate the SHASTA index (n=19) and APRI score (n=6).

Table 1 shows the characteristics of the 308 women by HCV status. Median age was 48 years and 67% were non-Hispanic black. The majority of women were overweight (median BMI of 25.6); nearly half reported current smoking; and nearly one third reported a history of injecting drugs. Excess alcohol use was uncommon with 45% reporting no alcohol use, 26% reporting light alcohol use, 14% reporting moderate alcohol use, and 13% reporting heavy alcohol use. The prevalence of HCV infection was 27%. Among the 84 HCV-infected women, 20% reported receiving anti-HCV treatment in the past, and there was no significant difference on the prevalence of low platelet counts (i.e., <150K) among the HCV-infected women receiving anti- HCV treatment compared to that among those without treatment (7/17 vs. 13/67; P=0.060). HCV-infected women also had higher median SHASTA and APRI scores (P ≤ 0.001); over half of HCV-infected women had significant fibrosis compared to 10% of HCV-uninfected women (P<0.001). Overall, the prevalence of significant liver fibrosis (defined as liver stiffness ≥ 7.1 kPa by TE) was 20%.

The characteristics of the HIV-infected women only, who accounted for 80% (240/308) of the study sample, were very similar to those of the entire sample. The prevalence of HIV/HCV co-infection was 24% (73/308). Among the HIV/HCV co-infected women, about half reported having AIDS compared with 33% among the HIV mono-infected women, (P=0.02). The prevalence of significant liver fibrosis was 22% among the HIV-infected women; about half of the HIV/HCV co-infected women and 10% of the HIV mono-infected women had significant liver fibrosis.

**Figure 1:**
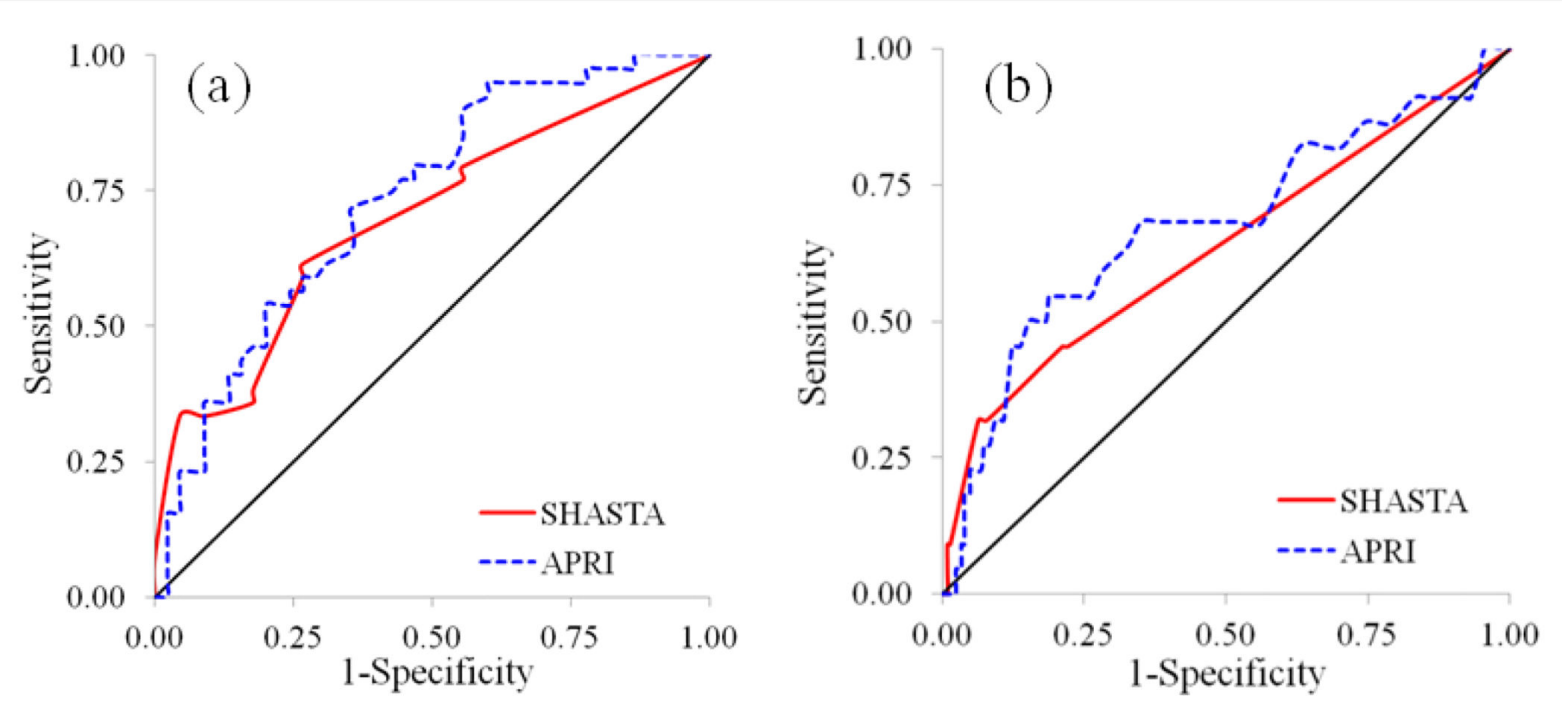
ROC curves for SHASTA and APRI among the total 308 women by HCV status together with the corresponding performace for the diagnosis of liver stiffness ≥ 7.1 kPa by TE : (a) 84 HCV+ and (b) 224 HCV−.

Figure 1 shows the ROC curves of SHASTA and APRI for the diagnosis of significant fibrosis among the total women by HCV status demonstrating little difference between SHASTA and APRI as predictors for significant fibrosis measured by TE. Both SHASTA and APRI had similar performance as predictors of predictors of significant fibrosis (≥ 7.1 kPa) among HCV+ women with an AUROC of 0.70 and 0.73, respectively, but performed less favorably as predictors of significant fibrosis among HCV− women with an AUROC of 0.64 and 0.67, respectively. Use of either SHASTA or APRI for the HCV+ women was associated with a positive predictive value of 65.7% and 69.6%, and a negative predictive value of 67.3% and 62.3% respectively. Among HCV− women, the positive predictive value of SHASTA and APRI was 35.0% and 16.2%, and negative predictive value was 92.6% and 91.4% respectively.

## Discussion

Accurate determination of liver fibrosis using non-invasive methods is important for the management of patients with chronic HCV infection, even in the modern era of effective and tolerable HCV medications with high rates of sustained virologic response. The great enthusiasm for the potential cure of patients with HCV infection using interferon-free regimens has been curbed by the high cost of the newly approved antiviral medications to treat HCV leading some to recommend delayed treatment of HCV in individuals without advanced fibrosis [20].

In this study, we found little difference between a direct and indirect serum biomarker of liver fibrosis, the SHASTA and APRI respectively, at predicting significant liver fibrosis (determined by TE) in HCV-infected women with or without HIV. This was an unexpected finding as we anticipated that the SHASTA would have more favorable test characteristics compared to the APRI, given that SHASTA includes HA, a marker of extracellular matrix deposition while the APRI consists solely of routine biochemical and hematologic measures which can easily be affected by HIV infection, related co-morbidities, and the concurrent use of medications in the setting of HIV. The role of HA in chronic inflammation and ongoing HIV replication may explain the limitations of SHASTA as a biomarker to predict liver fibrosis [21]. We also found that both biomarkers have significant associated misclassification that limits the utility of the test. In the past, studies have found that the highest positive and negative predictive values of these biomarkers are demonstrated with very early liver disease or minimal fibrosis, or the presence of cirrhosis. Our study demonstrated the relatively poor discriminatory characteristics of these assays even in a population with predominantly early stage fibrosis.

Determination of the extent of liver fibrosis therefore remains an important component of HCV management, including judicious use of health care resources. Selection of appropriate non-invasive modalities to measure fibrosis should take into account the patient’s clinical status, medications, co-morbidities, and body habitus. Identification of F0–1 and F4 disease by biomarkers such as APRI and SHASTA may be sufficient to guide treatment management decisions; those not at the extremes of no fibrosis or severe fibrosis should undergo further evaluation by TE or other more accurate composite biomarkers to avoid the 33–40% misclassification and underestimation of significant fibrosis, which may lead to potential treatment mismanagement.

## Figures and Tables

**Table 1:** Characteristics by HCV status among the 308 WIHS women.

	HCV- (n=224)	HCV+ (n=84)	*P* value
Age, yrs, median (IQR)	45.0 (38.9, 51.5)	53.1 (49.5, 56.6)	<0.0001*
Race/ethnicity, n (%)			0.22§
White non-Hispanic	40 (17.9)	10 (11.9)	
Black non-Hispanic	143 (63.8)	64 (76.2)	
Hispanic	27 (12.1)	8 (9.5)	
Other	14 (6.2)	2 (2.4)	
Alcohol use, n (%)			0.069#
Abstainer (no drinks)	100 (44.6)	39 (46.4)	
Light (<3 drinks/week)	61 (27.2)	19 (22.6)	
Moderate (3−13 drinks/week)	35 (15.6)	7 (8.3)	
Heavier (≥ 14 drinks/week)	24 (10.7)	17 (20.2)	
Smoker, n (%)			<0.0001#
Never	70 (31.3)	8 (9.5)	
Current	84 (37.5)	57 (67.9)	
Former	70 (31.3)	19 (22.6)	
Ever injected drug use, n (%)	28 (12.5)	67 (79.8)	<0.0001#
BMI, median (IQR)	25.6 (22.6, 28.7)	25.7 (21.9, 28.8)	0.83*
HIV status, n (%)			0.020#
Positive	167 (74.6)	73 (86.9)	
Negative	57 (25.4)	11 (13.1)	
HCV RNA × 10^3^ IU/ml, median (IQR)	/	2020 (651, 4020)	/
Ever HCV medication	/	17 (20.2)	/
AST, IU/L, median (IQR)	20 (16, 25)	38.5 (28.5, 71.5)	<0.0001*
ALT, IU/L, median (IQR)	16 (11, 23)	29 (20.5, 48)	<0.0001*
Platelets, × 10^3^/µl, median (IQR)	237.5 (203, 280.5)	193.5 (157.5, 228.5)	<0.0001*
Platelets<150 × 10^3^/µl, n (%)	9 (4.0)	20 (23.8)	<0.0001#
Albumin (g/L)	4.3 (4.1, 4.5)	4.0 (3.7, 4.3)	<0.0001*
HA, ng/mL, median (IQR)	22 (12.5, 38)	55.5 (31, 114)	<0.0001*
SHASTA, median (IQR)	0.02 (0.02, 0.02)	0.11 (0.02, 0.41)	<0.0001*
APRI, median (IQR)	0.26 (0.20, 0.33)	0.54 (0.38, 1.17)	<0.0001*
METAVIR stage using TE cutpoints, n (%)			<0.0001§
F0−1	202 (90.2)	45 (53.6)	
F2	15 (6.7)	13 (15.5)	
F3	3 (1.3)	10 (11.9)	
F4	4 (1.8)	16 (19.0)	

IQR: Interquartile Range; AST: Aspartate Aminotransferase; ALT: Alanine Aminotransferase; HA: Hyaluronic Acid; APRI: AST to Platelet Ratio Index

* Wilcoxon-Mann-Whitney test

# Chi-square test

§ Fisher exact test
